# Effects of foetal size, sex and developmental stage on adaptive transcriptional responses of skeletal muscle to intrauterine growth restriction in pigs

**DOI:** 10.1038/s41598-024-57194-9

**Published:** 2024-04-11

**Authors:** Y. Cortes-Araya, S. Cheung, W. Ho, C. Stenhouse, C. J. Ashworth, C. L. Esteves, F. X. Donadeu

**Affiliations:** 1 Division of Translational Bioscience, The Roslin Institute and Royal (Dick) School of Veterinary Studies, University of Edinburgh, Midlothian, UK; 2 Present Address: Department of Animal Science, Pennsylvania State University, State College, PA 16803 USA

**Keywords:** Intrauterine growth restriction (IUGR), Porcine, Skeletal muscle, Myogenesis, Foetal development, Developmental biology, Physiology

## Abstract

Intrauterine growth restriction (IUGR) occurs both in humans and domestic species. It has a particularly high incidence in pigs, and is a leading cause of neonatal morbidity and mortality as well as impaired postnatal growth. A key feature of IUGR is impaired muscle development, resulting in decreased meat quality. Understanding the developmental origins of IUGR, particularly at the molecular level, is important for developing effective strategies to mitigate its economic impact on the pig industry and animal welfare. The aim of this study was to characterise transcriptional profiles in the muscle of growth restricted pig foetuses at different gestational days (GD; gestational length ~ 115 days), focusing on selected genes (related to development, tissue injury and metabolism) that were previously identified as dysregulated in muscle of GD90 fetuses. Muscle samples were collected from the lightest foetus (L) and the sex-matched foetus with weight closest to the litter average (AW) from each of 22 Landrace x Large White litters corresponding to GD45 (n = 6), GD60 (n = 8) or GD90 (n = 8), followed by analyses, using RT-PCR and protein immunohistochemistry, of selected gene targets. Expression of the developmental genes, MYOD, RET and ACTN3 were markedly lower, whereas MSTN expression was higher, in the muscle of L relative to AW littermates beginning on GD45. Levels of all tissue injury-associated transcripts analysed (F5, PLG, KNG1, SELL, CCL16) were increased in L muscle on GD60 and, most prominently, on GD90. Among genes involved in metabolic regulation, KLB was expressed at higher levels in L than AW littermates beginning on GD60, whereas both IGFBP1 and AHSG were higher in L littermates on GD90 but only in males. Furthermore, the expression of genes specifically involved in lipid, hexose sugar or iron metabolism increased or, in the case of UCP3, decreased in L littermates on GD60 (UCP3, APOB, ALDOB) or GD90 (PNPLA3, TF), albeit in the case of ALDOB this only involved females. In conclusion, marked dysregulation of genes with critical roles in development in L foetuses can be observed from GD45, whereas for a majority of transcripts associated with tissue injury and metabolism differences between L and AW foetuses were apparent by GD60 or only at GD90, thus identifying different developmental windows for different types of adaptive responses to IUGR in the muscle of porcine foetuses.

## Introduction

Intrauterine growth restriction (IUGR) refers to the inability of an individual to attain their full genetic growth potential in utero, the most obvious features being distinctly low birth weight together with morphological characteristics of altered allometric growth, in addition to increased risk of neonatal morbidity and mortality^1^. IUGR is a significant health concern in both humans^2^ as well as domestic species, particularly the pig^3^. In addition to perinatal complications involving multiple body organs, IUGR individuals display impaired post-natal growth and are prone to a number of diseases later in life including metabolic and cardiovascular disorders^1^. In pigs, selective breeding for prolificacy in the absence of concomitant selection for uterine capacity has over the years resulted in an increase in birth weight variability within commercial litters, together with an increased incidence of IUGR^3^. IUGR pigs are defined by their weight at birth using various criteria such as a weight of less than 1.1 kg or less than two standard deviations of the mean body weight for age^4,5^. Based on this, most litters in genetically selected breeds contain at least one IUGR individual, with reported overall incidences of up to 25% newborns, attesting to the significance of IUGR as a health and welfare concern in this species^3^.

Although the triggers for IUGR are typically different between humans and pigs, placental insufficiency is a common feature in both species. Reduced placental function associated with reduced uterine capacity triggers an adaptive brain-sparing response in the IUGR foetus consisting in shunting of resources to brain and heart at the expense of less essential tissues such as skeletal muscle, digestive, renal, immune, neurological and reproductive. Selective resource allocation thus results in impaired development of those tissues, leading to life-long implications for body growth and physiological function^1^.

Together with reduced neonatal survival, poor growth and reduced carcass quality are primary causes of financial loss by the pig industry linked to the deleterious effects of IUGR^6^. IUGR causes a reduction in the number and size of skeletal myofibers in the foetus, in addition to changes in fibre type, that results in a permanent reduction in total muscle mass^7^. This, together with a tendency of affected animals to accumulate fat at the expense of lean tissue during post-natal growth^8,9^ leads to a significant reduction in meat production and quality. Reduced muscle fibre growth in both pig and sheep IUGR foetuses has been linked to impaired proliferation and differentiation of myoblasts^9–11^. In addition, we recently showed that mesenchymal progenitor cells isolated from IUGR piglets have an increased predisposition to generate fat at the expense of other tissue lineages such as bone and cartilage in vitro when compared to their non-IUGR littermates^9^.

Developmental programming as an adaptive response to IUGR in the pig is associated with widespread transcriptional and epigenetic dysregulation in foetal muscle, involving a myriad of developmental, tissue injury (i.e. associated with inflammation and immunity) and metabolic pathways, as shown by us and others^9,12–14^. Studies in sheep, another commonly used (experimentally induced) large animal model of IUGR, have shown that an increased inflammatory milieu in growth-restricted foetuses is induced by a catecholamine-mediated stress response to reduced resource availability, including hypoxia, to body tissues^15^. In parallel, extensive metabolic adaptation to limited resource availability occurs in foetal tissues such as muscle, consisting in a reduction in mitochondrial oxidative phosphorylation capacity with an increase in the use of fatty acids and amino acids relative to glucose as sources of fuel, increased lipid deposition^8,16^ and diminished protein accretion, among other changes^17–19^.

Importantly, significant within-litter variation in porcine foetal size has been reported from as early as gestational day (GD) 30 (gestational length, 115 days)^20^. Moreover, limited available data^8,21,22^ showed differences in muscle morphology and gene expression in growth restricted pig foetuses at GD45 or even earlier, suggesting that programming of the IUGR muscle phenotype indeed starts during early gestation. Yet, most knowledge on the effects of growth restriction on foetal tissue development comes largely from studies, in pigs or sheep, performed during late gestation, and usually considering single developmental time-points. A better understanding of the dynamic changes in key molecular pathways underlying adaptive responses of muscle to growth restriction at different stages of foetal development will be essential for developing effective strategies to ameliorate its detrimental effects on the livestock industry, most importantly pork, but also on life-long health in animals as well as humans. With this in mind, the objective of this study was to investigate gestational stage-dependent expression profiles in pig foetuses of selected genes that represent different functional categories (developmental, tissue injury and metabolism), and that, based on results of our previous analyses in late-gestation foetuses (GD90)^12,23^, may be involved in adaptive IUGR responses in skeletal muscle. To do this, we analysed muscle samples collected from sex-matched growth-restricted and non-growth restricted littermates at selected time-points during foetal development (GD45, 60 and 90) with the aim to obtain novel data on the molecular changes underlying the pathogenesis of IUGR in muscle.

## Results

### Littermate characteristics

Mean litter size and weights, as well as mean values for weight and crown-rump length (CRL) for AW and L littermates within each GD are shown in Table 1. As anticipated, mean weight (both absolute and adjusted to mean litter weight) and CRL values were lower for L littermates for all GDs. Significant effects or interactions involving Sex were not found for any endpoint, and therefore means averaged for males and females are shown in Table 1.

**Table 1:** Characteristics of experimental litters.

Endpoint		Gestational Day (GD)			Significant effect(s)
		45 (n = 6 litters)	60 (n = 8 litters)	90 (n = 8 litters)	
No. foetuses/litter (Mean ± SE)		16.50 ± 1.45	15.50 ± 1.16	14.00 ± 0.78	n.s
Litter weight (Mean ± SE)		20.82 ± 1.60	115.64 ± 7.25	674.90 ± 35.90	GD: *P* < 0.0001
Number of litters in which lightest foetus was male vs female		3 versus 3	4 versus 4	5 versus 3	n.s
Littermate weight (Mean ± SE)	AW	20.59 ± 1.49	115.39 ± 7.28	652.63 ± 41.67	GD: *P* < 0.0001 W: *P* < 0.0001
	L	16.58 ± 2.15*	86.50 ± 9.69**	386.69 ± 41.63**	
Littermate weight adjusted to mean litter weight (Mean ± SE)	AW	0.99 ± 0.01	1.00 ± 0.01	0.97 ± 0.03	W:*P* < 0.0001 GDxW: *P* = 0.006
	L	0.79 ± 0.06*	0.73 ± 0.04**	0.56 ± 0.04**	
Littermate CRL (Mean ± SE)	AW	70.33 ± 2.69	143.38 ± 9.10	241.38 ± 10.85	GD: *P* < 0.0001 W: *P* = 0.03
	L	62.00 ± 4.07*	134.75 ± 9.08^#^	213.00 ± 10.18*	

*AW* weight closest to the litter average, *L* Lightest, *CRL* Crown rump length, *GD* Gestational Day, *W* Weight, *n.s.* no significant effects, *P < 0.05, **P < 0.001, ^#^*P* < 0.1 (All symbols refer to comparisons between L and AW within each GD).

### Gene expression profiles of foetal muscle across gestational days, body weight and sex

To gain insight into the temporal dynamics of three key biological processes putatively involved in the pathogenesis of IUGR in muscle, namely development, tissue injury and metabolism^23^, we used RT-qPCR to quantify the expression of selected genes in each of those three categories. Genes for analysis (Suppl Table 2) were selected from a list of differentially expressed transcripts in porcine IUGR muscle on GD90^23^ based on (1) the availability of suitable primers and (2) their robust expression across tissue samples and developmental stages, as determined in preliminarily analyses. For completeness, the core myogenic regulators, PAX7, MYOD, MYOG and MSTN, were also included in the analyses. Moreover, for selected targets for which suitable anti-pig antibodies were available, namely the developmental gene ACTN3, and the metabolic genes, UCP3 and KLB (Supplementary Table 1), RT-qPCR results were validated using tissue immunochemistry on foetal samples from GD60 and GD90 (as indicated below, material harvested from GD45 foetuses was available for RNA analyses only).

### Developmental genes

**Figure 1:**
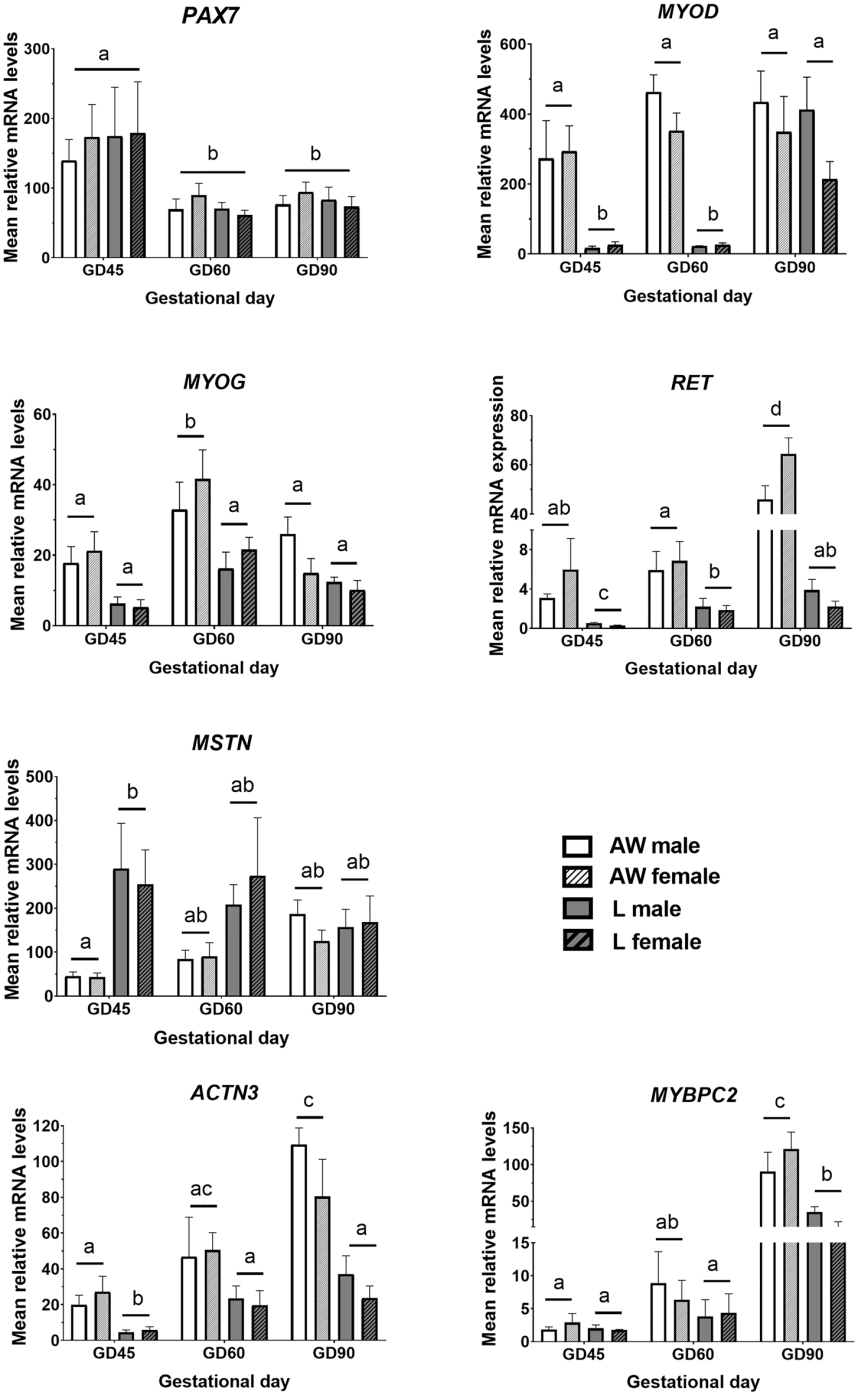
Relative mRNA levels (Mean ± SEM) of selected developmental genes in semitendinosus muscle of sex-matched foetal pairs comprising the foetus with body weight (BW) closest to the litter average (AW) and the lightest foetus (L) of pig litters at gestational day (GD) 45 (n = 6 litters), 60 (n = 8 litters) and 90 (n = 8 litters). There were signficant effects of GD, BW and/or an interaction for PAX7 (GD, *P* < 0.0001), MYOD (GD x BW, *P* < 0.0001), MYOG (GD, *P* < 0.0001; BW, *P* < 0.0001), RET (GD x BW, *P* < 0.0001), MSTN (GD x BW, *P* = 0.05), ACTN3 (GD, *P* < 0.0001; BW, *P* < 0.0001) and MYBPC2 (GD, *P* < 0.0001; BW, *P* < 0.003). Differences between group means are shown by different letters (abcd, *P* < 0.05).

Expression of RET, a receptor tyrosine kinase activated by GDFN family ligands, as well as the two muscle structural genes, ACTN3 and MYBPC2, were found to be downregulated in muscle from IUGR compared to normal weight foetuses on GD90 in our previous study^23^. Thus, expression of these development-associated transcripts were analysed together with (for completeness) the myogenic transcripts, PAX7, MYOD, MYOG and MSTN (Fig. 1). Mean PAX7 expression decreased distinctly between GD45 and GD60 but was not affected by foetal weight. In contrast, mean expression of MYOD, MYOG and RET transcripts were significantly reduced in L than AW littermates on one or more gestational dates; GD45 and 60 in the case of MYOD, GD60 for MYOG, and GD45, 60 and 90 for RET. The opposite expression profile was detected for MSTN, which transcript expression was significantly higher in L than AW littermates on GD45.

**Figure 2:**
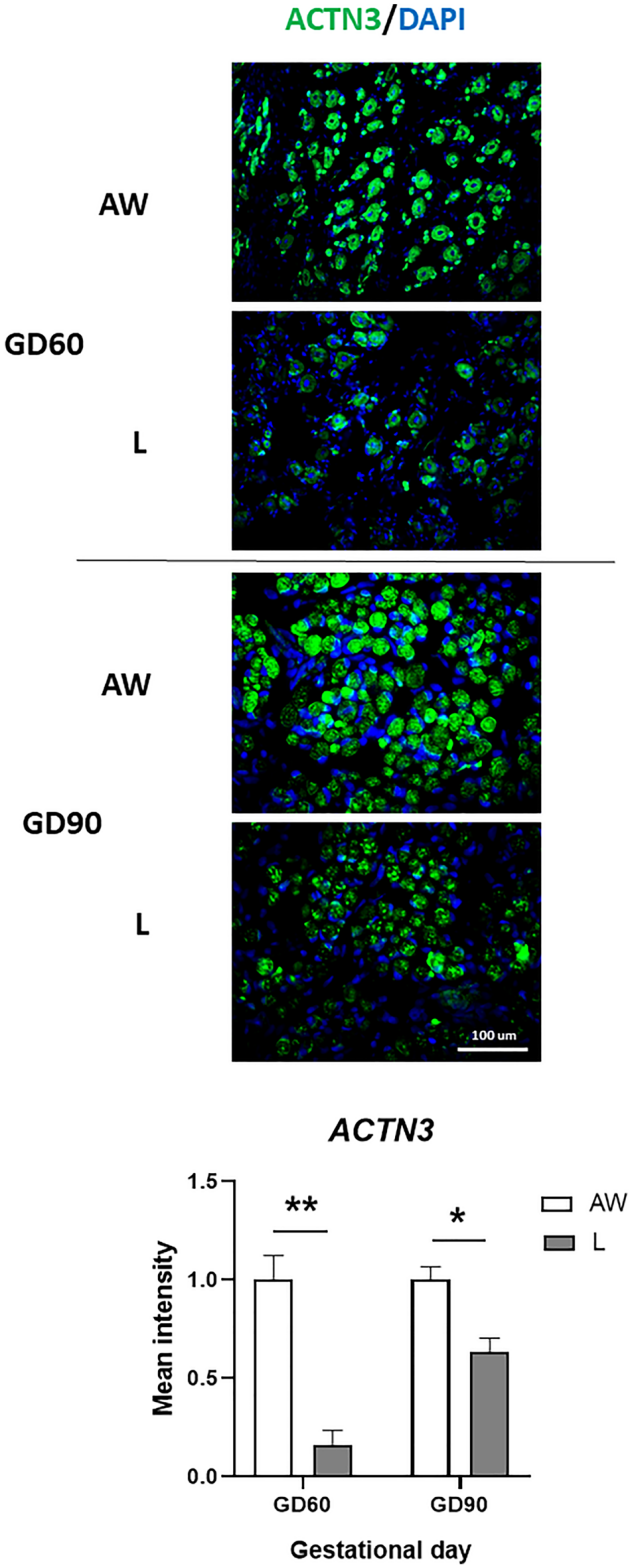
Representative images of semitendinosus muscle from the foetus with body weight (BW) closest to the litter average (AW) and the lightest foetus (L) of pig litters at gestational day (GD) 60 and 90 that were immunostained with ACTN3 antibody. Relative antibody signal (Mean ± SEM) is shown below for each GD (n = 3 litters), and differences between means are shown by * (*P* < 0.05) or ** (*P* < 0.01).

In addition, mean expression of ACTN3, quantified at mRNA and protein levels, was reduced throughout development in L littermates (Figs. 1 and 2), whereas MYBPC2 mRNA was reduced in the semitendinosus muscle from L foetuses on GD90 (Fig. 1). A significant effect or interaction involving Sex was not detected for any of the above transcripts.

### Tissue injury genes

**Figure 3:**
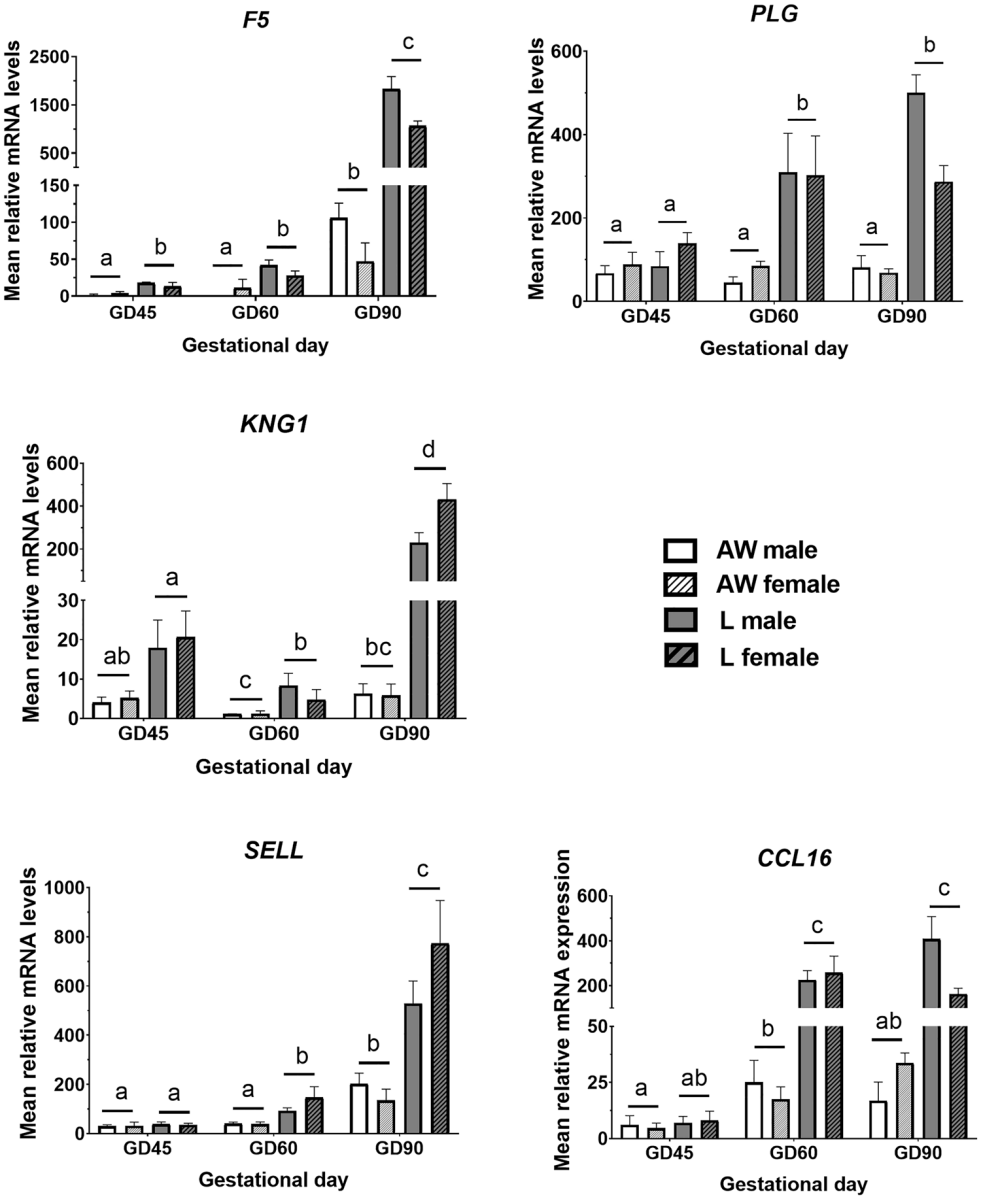
Relative mRNA levels (Mean ± SEM) of selected tissue injury-related genes in semitendinosus muscle of sex-matched foetal pairs comprising the foetus with body weight (BW) closest to the litter average (AW) and the lightest foetus (L) of pig litters at gestational day (GD) 45 (n = 6 litters), 60 (n = 8 litters) and 90 (n = 8 litters). There were signficant effects of GD, BW and/or an interaction for F5 (GD, *P* < 0.0001; BW, *P* < 0.0001), PLG (GD x BW, *P* < 0.005), KNG (GD x BW, *P* < 0.0001), SELL (GD x BW, *P* = 0.013), CCL16 (GD x BW, *P* < 0.0001). Differences between group means are shown by different letters (abcd, *P* < 0.05).

Changes in the expression of selected genes involved in tissue injury were determined, including coagulation factor V (F5), the plasmin precursor, PLG, the pro-coagulation/pro-inflammatory peptide precursor, KNG1, and two additional molecules involved in cell-mediated immune responses, namely, the leukocyte receptor, SELL, and the chemokine, CCL16 (Fig. 3). These were among a set of inflammatory/immune-related genes previously found to be upregulated in muscle from IUGR foetuses on GD90^23^. The present results showed that, in general, expression levels were, for all transcripts, similar between L and AW littermates at GD45, increasing progressively thereafter in L littermates so that levels were significantly higher than in AW littermates on GD60, and especially on GD90 (> threefold difference for all transcripts). There were no main effect or interactions involving Sex for any gene.

### Metabolic genes

In our previous study, we found that the largest category of differentially expressed transcripts in muscle between GD90 IUGR and normal weight foetuses corresponded to metabolism-related genes^23^. Accordingly, in the present study we selected a subset of those genes to determine the changes in their expression in relation to the development of the IUGR phenotype in foetal muscle. The genes selected for analyses corresponded to generic regulators of energy balance and tissue growth, and transcripts involved in specific nutrient metabolism pathways. The first class included the fibroblast growth factor 21 (FGF21) co-receptor, KLB, and the binding proteins, IGFBP1 and AHSG, whereas the second included genes specifically involved in metabolism of lipids (UCP3, APOB and PNLPA3), hexose sugar (ALDOB) and iron (TF). All of these were previously found to be upregulated in IUGR littermates at GD90, except UCP3 which was downregulated^23^.

**Figure 4:**
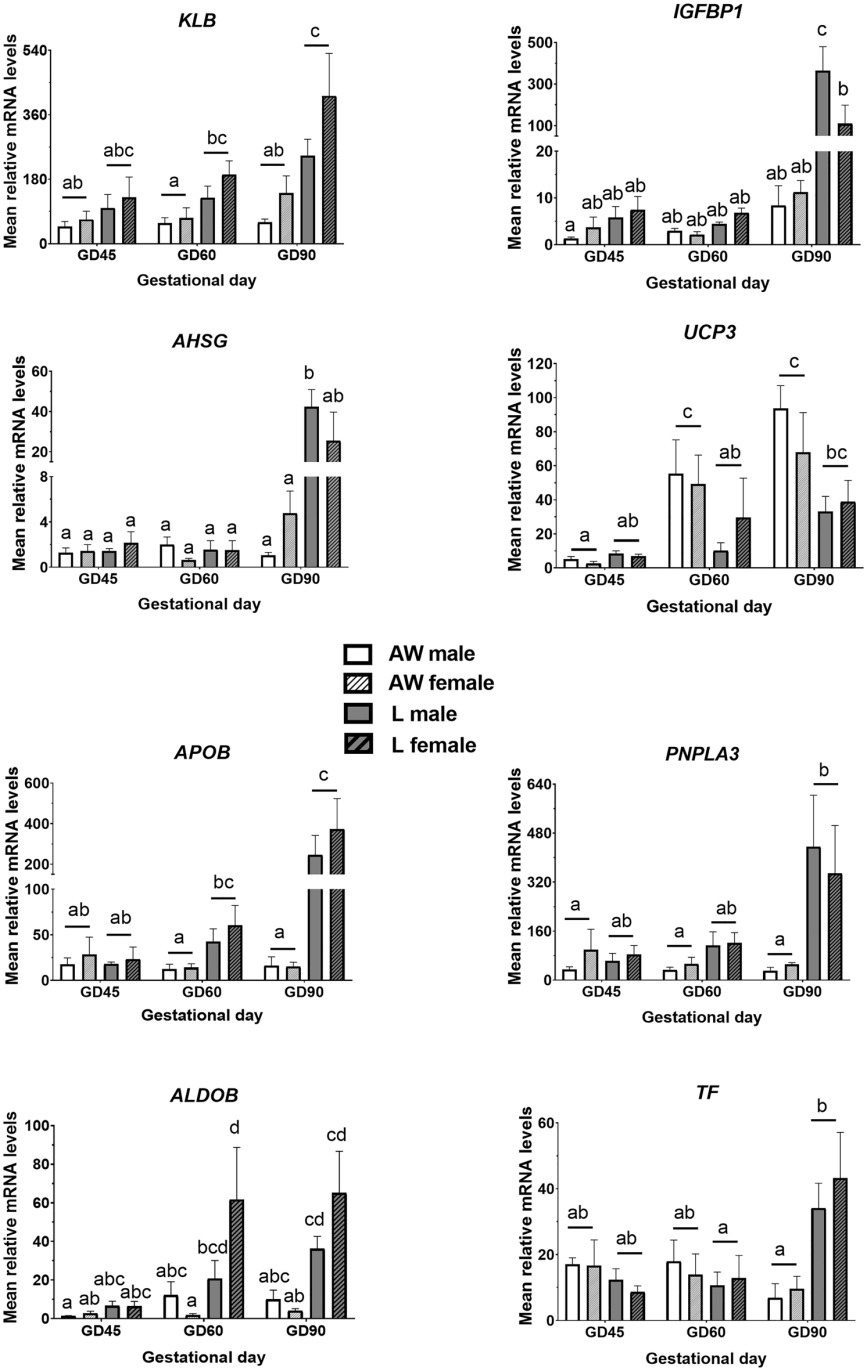
Relative mRNA levels (Mean ± SEM) of selected metabolism-related genes in semitendinosus muscle of sex-matched foetal pairs comprising the foetus with body weight (BW) closest to the litter average (AW) and the lightest foetus (L) of pig litters at gestational day (GD) 45 (n = 6 litters), 60 (n = 8 litters) and 90 (n = 8 litters). There were signficant effects of GD, BW and/or an interaction for KLB (BW, *P* < 0.0001), IGFBP1 (GD x BW x Sex, *P* = 0.035), AHSG (GD x BW x Sex, *P* = 0.048), UCP3 (GD x BW, *P* = 0.005), APOB (GD x BW, *P* = 0.003), PNLPA3 (BW, *P* < 0.0001), ALDOB (GD x BW x Sex, *P* = 0.039) and TF (GD x BW, *P* < 0.0001). Differences between group means are shown by different letters (abcd, *P* < 0.05).

**Figure 5:**
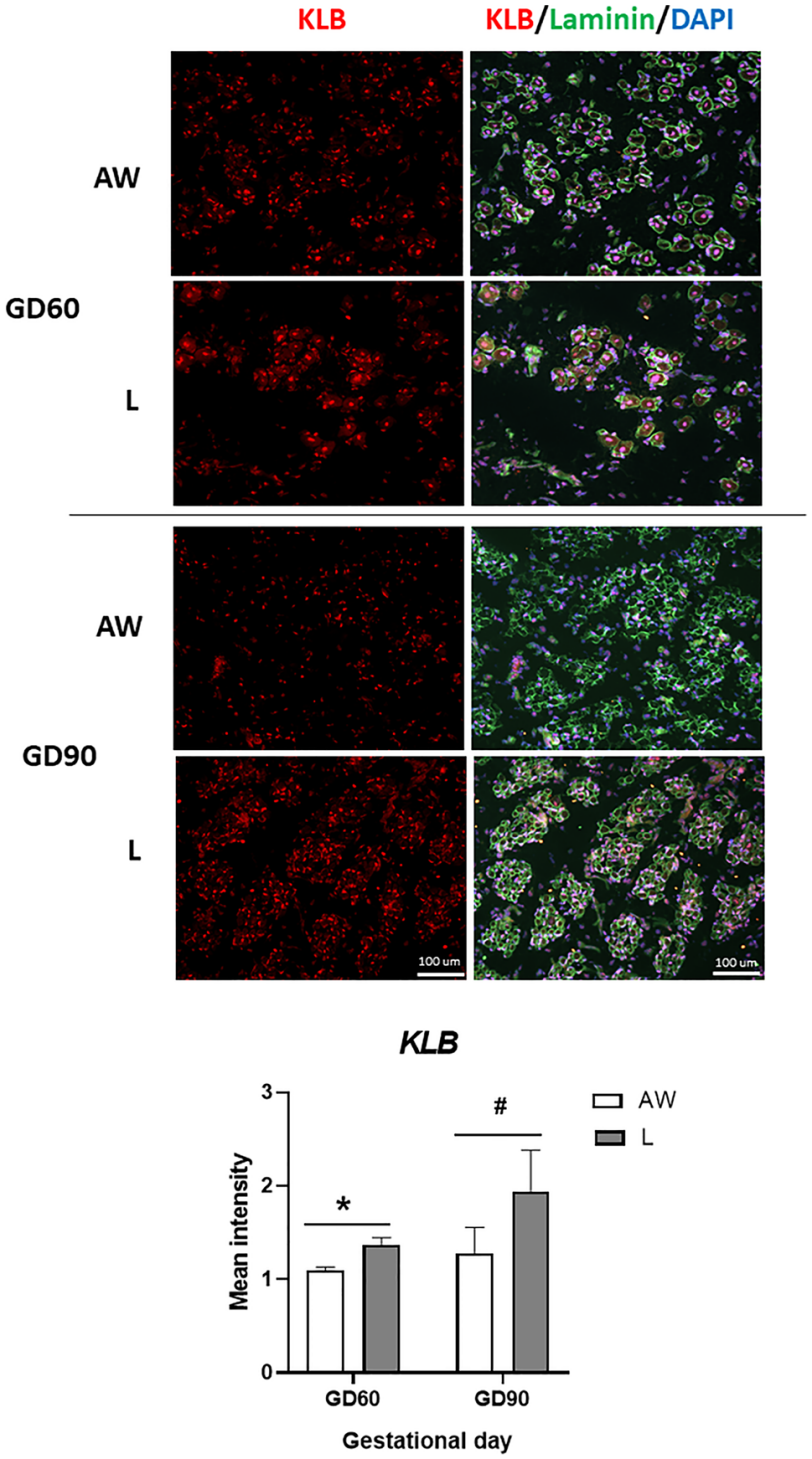
Representative images of semitendinosus muscle from the foetus with body weight (BW) closest to the litter average (AW) and the lightest foetus (L) of pig litters at gestational day (GD) 60 and 90 that were immunostained with antibodies against KLB and laminin. Relative KLB signal (Mean ± SEM) is shown below for each GD (n = 3 litters), and differences between means are shown by * (*P* < 0.05) or ^#^ (*P* < 0.1).

Expression of none of the metabolic transcripts was different between littermates on GD45 (Fig. 4). Expression of KLB mRNA was distinctly higher in L littermates on GD60 and, most prominently, on GD90 when compared to AW littermates (Fig. 4). This result was confirmed at the protein level by immunostaining of muscle sections with KLB antibody (Fig. 5). In contrast, transcript levels of IFGBP1 and AHSG both increased in expression in L relative to AW littermates but only at GD90, and these differences were restricted to males (Fig. 4).

**Figure 6:**
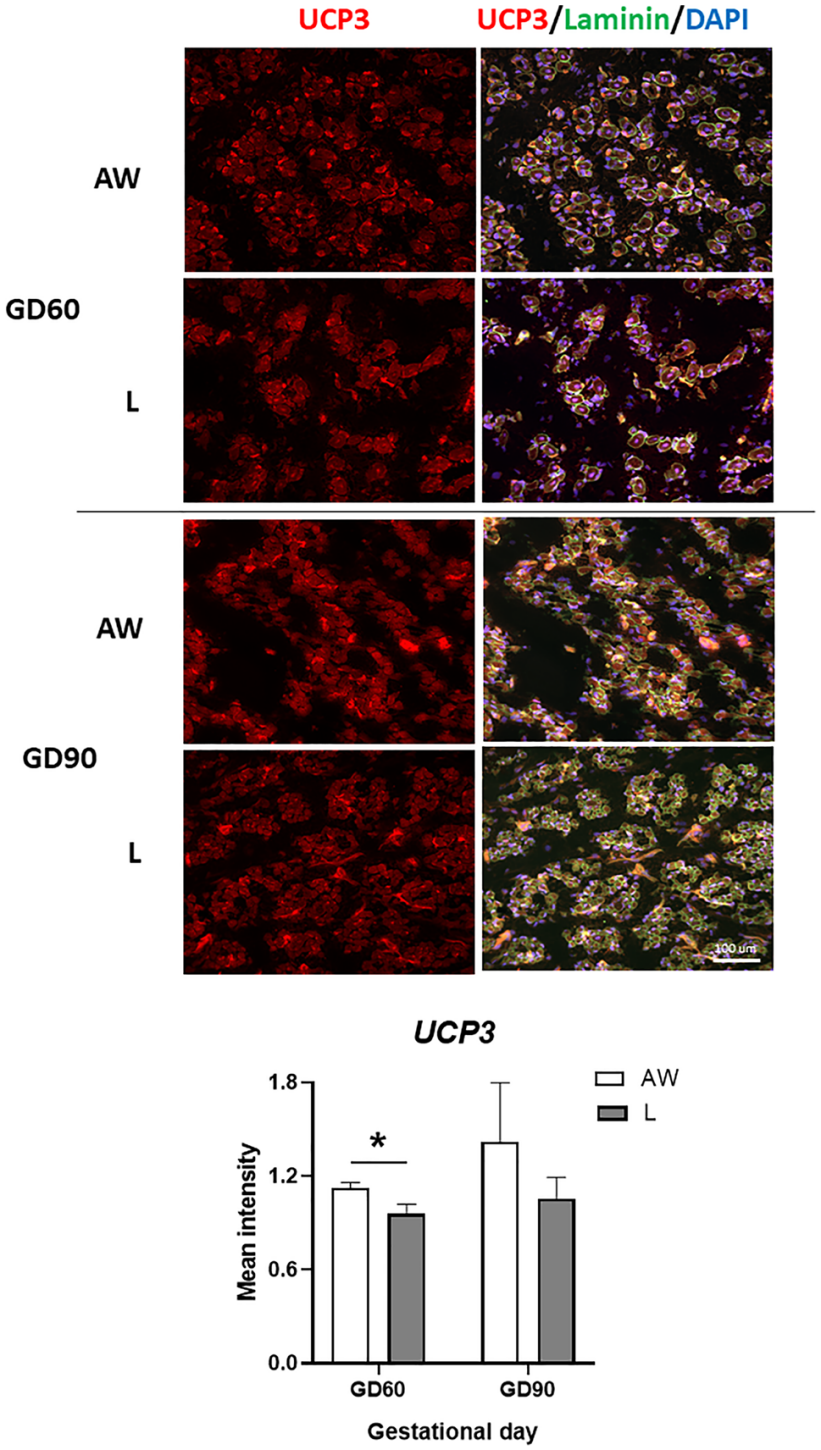
Representative images of semitendinosus muscle from the foetus with body weight (BW) closest to the litter average (AW) and the lightest foetus (L) of pig litters at gestational day (GD) 60 and 90 that were immunostained with antibodies against UCP3 and laminin. Relative UCP3 signal (Mean ± SEM) is shown below for each GD (n = 3 litters), and differences between means are shown by * (*P* < 0.05).

As for genes specifically involved in lipid metabolism, mean mRNA levels of UCP3 (one of few metabolism-related genes identified in our previous study as being downregulated in IUGR foetuses^23^) were significantly higher in AW than L foetuses on GD60 (Fig. 4). This was consistent with mean differences in protein levels determined by immunostaining with UCP3 antibody (Fig. 6). In contrast, mean mRNA levels of APOB were higher in L than AW foetuses on GD60 and GD90 (Fig. 4). The same trend was detected for PNLPA3 mRNA, although differences between littermates were significant on GD90 only. Interestingly, an interaction involving Sex was detected for ALDOB that resulted in significantly higher levels in L than AW littermates involving females only on both GD60 and GD90 Fig. 4. Finally, expression of TF was overall higher in L than AW littermates but only on GD90 Fig. 4.

## Discussion

Limited knowledge is available on the molecular mechanisms driving altered skeletal muscle development in growth-restricted pigs, with only a few studies focusing on early developmental events^24,25^. Moreover, the effects of foetal sex have often been overlooked in previous large animal studies, and must be included to fully understand the mechanisms involved in developmental programming^26,27^. Building on the results of previous transcriptome-wide profiling in late-stage pig foetuses^12,23^, the present study reports on the expression dynamics of different transcripts involved in key aspects of the adaptive response of foetal muscle to IUGR. Importantly, expression changes were characterised between littermates differing in weight and sex, and across critical stages during hyperplasic muscle development. These included primary myogenesis (GD45), as well as the initiation (G60) and completion (GD90) of secondary myogenesis^22^. By providing information on the temporal aspects of transcriptional events related to specific signalling pathways during muscle development, the study provides novel insight into the pathogenesis of IUGR which could lead to identification of potential targets for early intervention to ameliorate its effects on pig health and productivity.

Differences observed in the expression of key myogenic regulators suggest that the reduced muscle growth in L littermates may not be attributed to differences in the abundance of progenitor PAX7-expressing cells but to a reduction in the proportion of such cells entering the myogenic differentiation programme. This was evidenced by the reduced expression, from as early as GD45, of the myogenic regulatory factors (MRFs), MYOD and (to a lesser extent) MYOG, as well as RET, which reportedly acts to maintain proliferation and prevent early differentiation of myoblasts^28^. In agreement with these results, decreased MRF expression in muscle was detected as early as GD30 in response to foetal overcrowding in pigs^21^. However, another study^24^ failed to detect differences in MYOD between the smallest and largest littermates between GD36 and GD86. A developmentally programmed reduction in the differentiation ability of muscle progenitor cells in IUGR is also supported by various studies in both sheep^10,29^ and pig^23^ demonstrating that cells from IUGR foetuses have reduced capacity to produce myotubes in vitro, in association with reduced expression of MYOD and MYOG^29^.

MSTN is a key regulator of muscle growth which acts by supressing satellite cell activation and myoblast differentiation, putatively through transcriptional repression of MRFs, and promotion of protein degradation^30^. A negative relationship between MSTN and MYOD/MYOG expression profiles in AW and L littermates during GD45 and GD60 suggests an active role of MSTN in restricting primary and potentially also secondary myogenesis in L littermates. A previous study^24^, reported higher expression of MSTN in semitendinosus muscle of the smallest pig littermates but only after GD60, whereas MSTN expression was actually reduced in the same muscle in late-stage sheep IUGR foetuses^29^, as well as in longissimus dorsi from low birth weight piglets^31^. Clearly, the role of MSTN in the IUGR muscle phenotype is complex and will need clarification in future studies. Nonetheless, overall, our data on temporal profiles of myogenic regulators indicate that a reduction in the commitment of progenitor cells to the myogenic programme underlies impaired muscle formation from early stages of development (primary myogenesis) in growth restricted pig foetuses.

IUGR has been associated with changes in the relative abundance of different fibre types (slow vs fast-twich) in foetal muscle, which are involved in the metabolic adaptation to IUGR^32,33^. Our results of reduced expression of the type-II (Fast-Twitch)-associated genes, ACTN3 and MYBPC2, in L muscle are consistent with previous results from proteomics analyses in foetal IUGR muscle in pigs^34^, and with the finding that IUGR muscle contains reduced numbers of secondary fibres, from which most type II fibres derive^22^. Taken together, our results are consistent with both quantitative and qualitative changes in muscle formation occurring as an adaptation to IUGR from early (primary) myogenesis.

Studies conducted in various species have provided evidence that systemic inflammation occurs in IUGR as an adaptive response to the stress induced by hypoxemia and nutrient deficiency^23,35–37^. However, to our knowledge, the temporal dynamics of this inflammatory response during foetal development has not been thoroughly investigated. Previous studies showed that inflammation-related genes are upregulated in muscle of IUGR/low weight pig foetuses on GD70^14^ and GD90^23^. Our results with a small subset of genes associated with coagulation and cell-mediated immunity expand on those earlier findings by showing that differences in inflammatory milieu between L and AW foetuses, albeit modest, can be detected as early as GD45 (as demonstrated by significant differences in F5 levels between littermates), and that they distinctly increase thereafter on GD60 and, particularly, GD90. This novel observation suggests that an adaptive inflammatory response to IUGR may already occur before mid-pregnancy in pig foetuses. Moreover, our results of a progressive increase in the expression of inflammatory markers between GD45 and GD90 are consistent with an increase in resource demand by the foetus (and therefore in the stress response associated with growth restriction) as the second half of pregnancy progresses, coupled with a simultaneous increase in the functional maturation of the immune system in the porcine foetus^38^.

Based on the limited set of representative transcripts analysed in our study (Fig. 4), a metabolic adaptive response to nutrient deficiency in growth restricted pig foetuses took place only after GD45, and gradually increased between GD60 and GD90, coinciding with the period when skeletal development is highest^22^. Although extensively studied in relation to metabolic homeostasis and obesity in the adult^39^, the role of FGF21 in the foetus and specifically in the pathogenesis of IUGR has not been established. We have shown previously that an increase in the expression of the FGF21 co-receptor, KLB, locally mediates the adaptive response to IUGR by restricting muscle formation in GD90 pig foetuses^23^. The present results of higher expression levels of KLB in muscle of L foetuses suggest that FGF21 signalling may be involved in metabolic programming already by GD60 in the IUGR foetus, justifying future study to better understand the involvement of this important metabolic regulatory pathway on foetal growth.

Increased utilization of fatty acids as an energy source is a key feature of metabolic programming in the IUGR foetus^14,40^. Our findings indicate that alterations in fat and sugar metabolism occur as early as GD60 in L foetuses, as shown by differences in the expression of UCP3, a protein involved in transport of fatty acids from/into the mitochondria^41^, APOB, a major lipoprotein component, and the glycolytic enzyme, ALDOB. These results align with previous reports of higher levels of plasma cholesterol as well tissue fatty acid synthesis and ALDOB expression in low-weight pig foetuses already by GD63^13,42^. Moreover, our results suggest that lipid usage by muscle markedly increases in growth-restricted foetuses during the second half of gestation, as suggested by much higher expression levels of APOB and the triacylglycerol lipase, PNPLA3, in L than AW piglets by GD90.

The finding that IGFBP1 and AHSG were expressed at higher levels in muscle of L compared to AW foetuses on GD90 is consistent with reports of elevated blood levels of these two factors in human IUGR cord blood^43,44^, as well as, in the case of AHSG, in plasma of neonate IUGR piglets^45^, in which a negative correlation of AHSG levels with post-natal growth potential was also reported. In fact, it has been postulated that IGFBP1 and AHSG respond to tissue hypoxia to modulate nutrient availability as part of the adaptive growth responses to IUGR^46,47^. Moreover, the liver provides the primary source of IGBP1 and AHSG in the foetus, and in the context of IUGR in the pig, liver-derived IGBP1 and AHSG may already be involved in foetal adaptive growth responses before GD90^44,46^. In that regard, the higher expression of IGFBP1 and AHSG observed in muscle of L foetuses on GD90 may reflect a response to increasing nutrient requirements locally by the rapidly growing tissue or, in addition/alternatively, in response to rapidly increasing resource demands systemically by the growing foetus above those than can be met by production of IGFBP1 and AHSG by the liver alone.

A notable observation in our study was that, as was the case for another metabolism-related gene, ALDOB, expression of both IGFBP1 and ASHG in muscle followed a sexually dimorphic expression pattern. In the case of IGFBP1 and ASHG, an increase in expression in L foetuses on GD90 was significant in males only. This observation suggests a more pronounced adaptive response to growth restriction in males, and this is consistent with the observation that males are more sensitive than females to the disruptive effects of IUGR on growth and metabolism, accounting for reduced postnatal survival and increased disease risk later in life (reviewed in^48^). In agreement with our results, reduced expression of AHSG in skeletal muscle of female compared to male foetuses in Iberian pigs (a ‘thrifty’ breed) but not in crossbred pigs, were reported in a recent study^14^. Interestingly, increased AHSG levels have been shown to be associated with the development of insulin resistance and metabolic disease^49^, which incidence is increased in male IUGR individuals.

For some developmental stages, only 3 individuals from either sex were available for analyses in our study. Although a limitation, this did not preclude the identification of significant sex-dependent effects, specifically for 3 of the 20 genes analysed. This is in agreement with previous transcriptome-wide studies which found limited effects of sex on muscle expression profiles in pig foetuses of different sizes^14,50^. Still, we recognise that because of small sample sizes we may have failed to detect mild effects on some of the genes analysed, and this should be determined in future studies.

In summary, expression profiling of skeletal muscle from L and AW littermates of different sexes across foetal development (GD45, 60 and 90), revealed differences in the temporal patterns of different functional gene categories, namely developmental, tissue injury-related and metabolic. In particular, marked differences in the expression of several key myogenic regulators and structural genes indicative of reduced myogenesis in L foetuses were present by GD45, whereas clear differences in the expression of several tissue injury genes, namely an increase in coagulation and cell-mediated immunity transcript levels, were mainly detected by GD60, with a further increase at GD90. Similarly, based on changes in levels of transcripts involved in metabolic regulation, as well as those related to lipid, sugar and iron metabolism specifically, an adaptive metabolic response in L foetuses occurred only by GD60 and was most prominent at GD90. Finally, effects of sex on gene expression profiles were only observed for ALDOB, IGFBP1 and AHSG, the latter two suggesting a more pronounced adaptive response to growth restriction occurred in males. In conclusion, results from analyses of a limited number of representative transcripts in pig fetuses indicate that changes in different types of adaptive responses occur sequentially during development of the IUGR muscle phenotype in the pig fetus, with early effects on muscle growth (before GD45) preceding the establishment of adaptive inflammatory and metabolic responses, both of which increase in intensity during the second half of pregnancy, alongside other effects suggestive of sex-biases in IUGR adaptation that favour females. From a clinical point of view, these results identify gestational stage and foetal sex as important factors to take into account when considering the outcomes and effectivity of interventional strategies aimed at preventing or ameliorating the effects of IUGR in the developing foetus.

## Methods

### Experimental animals and sample collection

All procedures were performed with approval from The Roslin Institute (University of Edinburgh) Animal Welfare and Ethical Review Board and in accordance with the U.K. Animals (Scientific Procedures) Act, 1986. The study was carried out in compliance with the ARRIVE guidelines.

Foetal tissues were collected from Large White X Landrace gilts on GD45 (n = 6), GD60 (n = 8) or GD90 (n = 8) that were euthanized as part of a separate study^23^. Briefly, pregnant gilts were euthanized using sodium pentobarbitone 20% w/v (Henry Schein Animal Health, Dumfries, UK) at a dose of 0.4 ml/kg by intravenous injection. Each gilt was hysterectomized by mid-ventral incision and the gravid uterus was placed in a dissecting tray and opened to expose each foeto-placental unit. For each litter, all foetuses were extracted and individually weighed, after which the lightest littermate (L) together with the gender-matched littermate with weight closest to the litter average (AW) were selected. A skeletal muscle sample from the hind limb area corresponding to the semitendinosus was snap-frozen in liquid nitrogen before storage at − 80 °C for later RNA analyses. A piece of semitendinosus from the opposite limb was transported on dry ice to the laboratory where it was embedded in Tissue-Tek OCT Compound (Miles Laboratories, Clifton, NJ, USA), frozen in isopentane cooled over dry ice, and stored at − 80 °C for immunochemistry analyses. Due to the limited amount of tissue available from GD45 fetuses no samples were harvested for immunochemistry in that group.

### Tissue immunochemistry

The Cryostat (Leica CM 1900, Leica Biosystems, Stantonbury, UK) was set at − 20 °C and 10 μm sections were collected on pre-warmed positively charged microscopes slides (Thermo Fisher Scientific, Waltham, MA, USA) and left to dry at room temperature and stored at − 80 °C.

Frozen tissue samples were fixed by submerging the slides in cold analytical grade acetone on ice for 20 min followed by incubation with acetone and methanol (1:1) at room temperature (RT) for 10 min. Slides were washed with phosphate buffered saline (PBS) three times and then incubated with Trident universal protein blocking reagent (GeneTex, Inc, CA, USA) for 1 h. Slides were then washed three times with PBS and incubated with primary antibody (Suppl. Table 1) reconstituted in antibody diluent buffer (Thermo Fisher Scientific). Slides were incubated in a humidified chamber overnight at 4 °C in the dark and then washed three times with PBS before incubation with fluorescent-conjugated secondary antibody (Suppl. Table 1) for 1 h at RT. Sections incubated with secondary antibody only were used as negative control (Suppl. Figure 1). All samples were then washed three times with PBS followed by mounting in Fluoroshield with DAPI (Sigma-Aldrich, St. Louis. MO, USA).

Slides were visualised using the Leica DMLB upright fluorescent microscope. From each slide, at least three different sites were randomly chosen for image acquisition using a 20X objective lens. All images were acquired with short exposure to avoid photobleaching of fluorophores. Image acquisition and analysis were performed using Fiji software (https://fiji.sc/#). All images were converted to an 8-bit format followed when required by adjusting of Laminin intensity threshold to reveal cell border and shape. The selector tool was then used to manually identify each individual cell or cell cluster. Fluorescent intensity values were measured and normalized against the corresponding DAPI value using the region of interest (ROI) manager tool.

### RNA analyses

Fragments of whole skeletal muscle (30 mg) were homogenized in RNABee (AMS Biotechnology, Abingdon, UK) in Lysing Matrix D tubes (MP Biomedicals, Illkirch, France), and extracted according to manufacturer's instructions followed by transfer to a RNeasy Mini Spin column and treatment with RNase-free DNase (Qiagen, Manchester, UK). RNA was quantified using a Nanodrop ND-1000 (Labtech International Ltd., Heathfield, UK) and processed for 2-step qPCR. Complementary DNA (cDNA) was prepared from 500 ng RNA using SuperScript III reverse transcriptase (Thermo Fisher Scientific) following the manufacturer’s instructions. Quantitative PCR was then performed on a MX3005P system (Stratagene, La Jolla, CA, USA) using Sensi-FAST SYBR Lo-ROX (Bioline, London, UK), as per manufacturer instructions, and porcine-specific primers (Supplementary Table 2). A standard curve made of serial dilutions of pooled muscle cDNA was run on parallel. Test samples together with standards and no template and reverse transcriptase controls were all run in duplicate. Data were analyzed using MxPro Software. Expression levels for each sample were normalised to the average expression of the TOP2B, RPL4 and HPRT1. These three normalisers were identified based on stable expression on foetal muscle samples using geNORM V3.5 (Ghent University Hospital, Centre for Medical Genetics).

### Statistical analysis

All statistical analyses were performed using Minitab 20 Statistical Software. For each endpoint, data normality was assessed by Kolmogorov–Smirnoff test (*P* > 0.01) and log10 transformation was applied where required. ANOVA was then used to determine significant effects of littermate Weight, GD and Sex, and their interactions, using Litter as co-variate, followed by post-hoc Tukey or Bonferroni tests. Significance was considered at *P* ≤ 0.05, whereas differences with *P* < 0.1 were taken as approaching significance.

### Supplementary Information

Supplementary Figure 1.

Supplementary Tables.

## Data Availability

All data is available within the manuscript.
