# Supplementary figures and images for: Effects of foetal size, sex and developmental stage on adaptive transcriptional responses of skeletal muscle to intrauterine growth restriction in pigs
Source: Sci Rep. 2024 Apr 11;14:8500. doi: 10.1038/s41598-024-57194-9 (PMC11009347; doi:10.1038/s41598-024-57194-9)

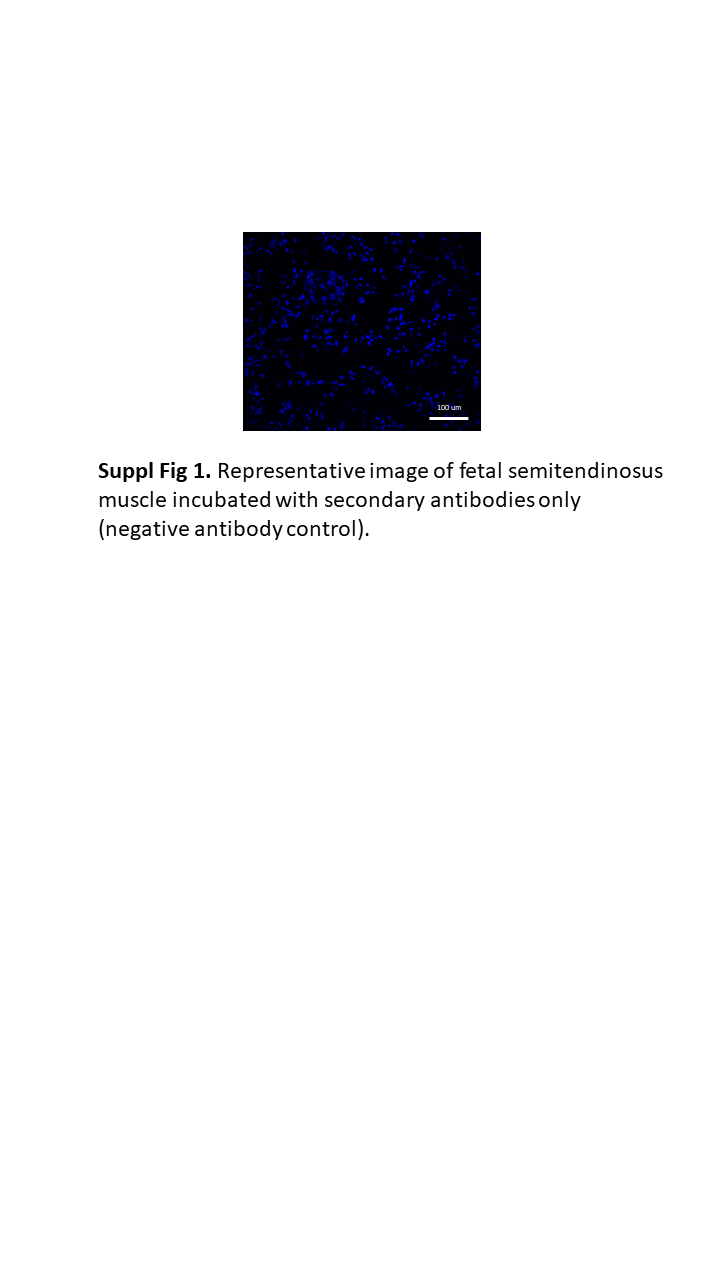

Supplement: Supplementary file 1 — Supplementary Figure 1. [file 41598_2024_57194_MOESM1_ESM.tif]
